# Endovascular management in venous disease

**DOI:** 10.4103/0971-3026.40303

**Published:** 2008-05

**Authors:** John D'Souza, Abhay K Kapoor, VS Bedi

**Affiliations:** Department of Radiodiagnosis, INHS Asvini, Mumbai - 400 005, India; 1 Vascular Surgery, R and R, Delhi - 110 010, India

**Keywords:** Deep vein thrombosis, stenting, thromboaspiration, thrombolysis

## Abstract

**Background:**

Deep vein thrombosis (DVT) and pulmonary embolism (PE) are under-recognized entities and can be often difficult to diagnose. Occasionally, clinical symptoms, such as pain and swelling, are non-specific or absent. If left untreated, the thrombus can get dislodged and migrate to cause life-threatening pulmonary embolism.

**Materials and Methods:**

Twenty patients presenting with limb edema and local increase in temperature were screened with color Doppler. Of the 20 cases, three were treated for upper limb venous disease and 17 were treated for lower limb disease. Cases detected to be positive for DVT were taken up for diagnostic venography using DSA. On confirmation, these patients were offered endovascular management viz. percutaneous recanalization by thromboaspiration or thrombolysis or a combination of both. Patients with residual stenosis or vessel occlusion were treated with balloon angioplasty/stent placement. The patients were assessed with a color Doppler study at 6, 12, 24, and 36 months.

**Results:**

Of the 20 cases, four patients underwent thromboaspiration, eight underwent thrombolysis, eight were treated with angioplasty, and seven patients required stent placement. Technical success was achieved in 95% (19 of 20) of the cases treated with thrombolysis and/or venous stent placement. One patient had complete occlusion of the vein preventing passage of catheter and thromboaspiration. Three patients were treated for upper limb disease and on follow-up studies, all three remained patent while of the 16 patients treated for lower limb disease, 13 remained patent giving a success rate of 81.25%. No complications occurred in any of the patients. Two patients developed a stent thrombus at 48 and 72 h respectively, both of which were recanalized by balloon angioplasty.

**Conclusions:**

In venous thrombosis, color Doppler helps in the detection and morphological analysis of disease, while venography confirms and assists in managing venous thrombosis using interventions. Endovascular interventional management includes percutaneous recanalization by thromboaspiration or thrombolysis or a combination of both. Patients with residual stenosis or vessel occlusion vessel can be further treated with balloon angioplasty/stent placement.

## Introduction

Venous thrombosis commonly leads to pulmonary embolism (PE) and accounts for 90% of all cases of acute PE.[1] Lower extremity deep vein thrombosis (DVT) is commoner than upper limb disease with a prevalence rate of 1 per 1000 population.[1] The various causes of lower limb venous disease include immobilization, coagulation disorders, post-Cesarean surgery, and the May-Thurner syndrome,[2] while upper limb disease is most commonly caused by long-standing indwelling catheters, trauma and following a biopsy or central line insertion.

Venous stasis, vessel wall injury and a hypercoagulable state, form the classic triad of predisposing factors for the development of DVT. This triad was first described by Virchow in 1856.[3] Any pathology that affects either of these factors may produce DVT. With anticoagulation alone, as many as 75% of patients with symptomatic DVT present 5-10 years later with PE.[4,5] Endovascular management of DVT shows a good long-term patency rate and has scored over systemic intravenous (IV) thrombolysis, which often has a higher rate of reocclusion and increased incidence of bleeding complications.[6]

The objective of this study was to evaluate the efficacy, complications and long-term effects of endovascular management in patients with thrombosed veins using thromboaspiration, thrombolysis or venous stent placement. Over a 3-year period, 20 patients who presented with limb edema or locally raised temperature were investigated.

Until the 1980s, venography was the standard investigation for the diagnosis of DVT. This procedure is now uncommonly performed because of patient discomfort and the increasing accuracy of USG and color Doppler. Venography is now used in very specific situations, especially when absolute determination of the presence and extent of thrombus is needed, usually prior to endovascular management.[7]

## Materials and Methods

A prospective longitudinal study was conducted over a period of approximately 3 years in a population of 20 patients, who were treated with endovascular techniques, symptomatic with lower limb or upper limb edema or with locally raised temperature. Evaluation included technical success, complications and the long-term efficacy for each modality of therapy.

### Inclusion criteria

Patients diagnosed with DVT on color Doppler were treated with heparin. Those not responding within 72 h were further evaluated with a repeat color Doppler followed by venography. On confirmation, these patients were offered endovascular management, which included percutaneous recanalization by thromboaspiration or thrombolysis or a combination of both. Patients with residual stenosis or vessel occlusion were treated with balloon angioplasty/stent placement.

### Equipment

Pre- and post-procedure USG was carried out with commercially available scanners (Logic 400 and 500; Wipro GE, India), equipped with 3.5, 5, and 10 MHz transducers. The interventional procedures were carried out on a standard digital subtraction angiography machine (Polystar 1000 mA; Siemens, Germany).

### Procedure

#### Venography:

The deep veins of the lower limbs were accessed by performing a USG-guided puncture of a popliteal vein, followed by the placement of an access sheath. Venography was performed through the sheath and depending on the extent of the thrombus, the patient was treated with catheter-directed thrombolysis (CDT), mechanical thrombectomy, angioplasty and/or stenting of the venous obstruction. Venography of the upper limbs was performed via the antecubital vein, followed by the placement of an access sheath for further interventions.

#### Catheter-directed thrombolysis:

Fluoroscopic guidance was used to place infusion catheters or devices[8,9] though which recombinant tissue plasminogen activator (r-tPA) was infused. A 20-mg bolus was given, followed by an infusion of 30 mg over 6 h, to maximize the exposure of the thrombus to the thrombolytic.

#### Percutaneous mechanical thrombectomy:

This was performed using a 6-F thromboaspiration catheter (Oasis; Boston Scientific/MediTech, Watertown, MA, USA), which utilizes the Venturi principle and acts by mobilizing the fresh clot and subsequently removing it by suction.

#### Balloon angioplasty:

This procedure was used mainly as an adjunct to stent placement. Veins have high elastic recoil, which negates the advantage gained by balloon dilatation alone. As a result, stenting is invariably required. However, pre-dilatation of the affected segment using an angioplasty balloon helps in placement of a stent thereafter.

#### Venous stenting:

After thrombolysis or thrombectomy, patients with residual stenosis required stent placement.[10,11] Self-expanding Nitinol stents were used. If the stents needed to be placed at a level below the inguinal ligament, then stent insertion was terminated above the insertion of the greater saphenous vein.

### Post-procedure management

Post-stent placement patients were started on long-term antiplatelet agents, e.g. aspirin (75 mg/day) or clopidogrel (75 mg twice/day), for approximately 2 year. Based on routine anticoagulation regimes for DVT, Warfarin was started with the aim of keeping the International Normalized Ratio (INR) at 2-3; this was maintained for 6 months.

Color Doppler was performed on the day following the procedure to ensure stent.

## Results

**Figure 1:**
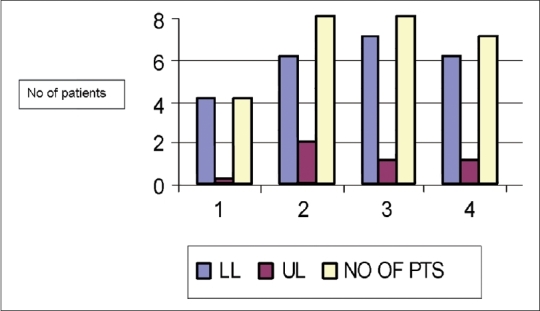
Bar diagram shows the distribution of the endovascular procedures performed for the management of various venous diseases. 1-Thromboaspiration; 2-Thrombolysis; 3-Angioplasty; 4-Stent placement; LL-Lower limb; UL-Upper limb

**Figure 2(A, B):**
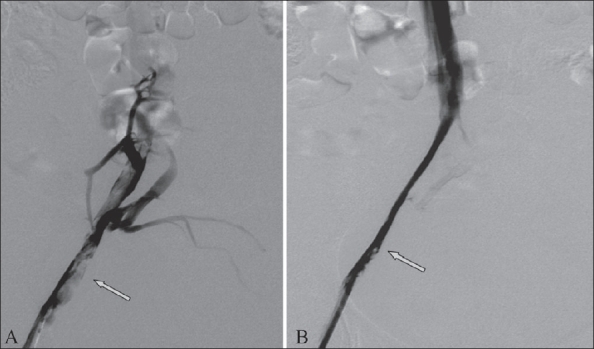
A 34-year-old patient presented with features of DVT in the right leg, following a cesarean section. Post-Doppler venography (A) shows a thrombus (arrow) in the common femoral vein (CFV), external iliac vein (EIV) and common iliac vein (CIV). Thrombolysis was performed using an OASIS thrombolytic catheter. Post-thromboaspiration check venography (B) shows good clearance of thrombus (arrow)

**Figure 3(A, B):**
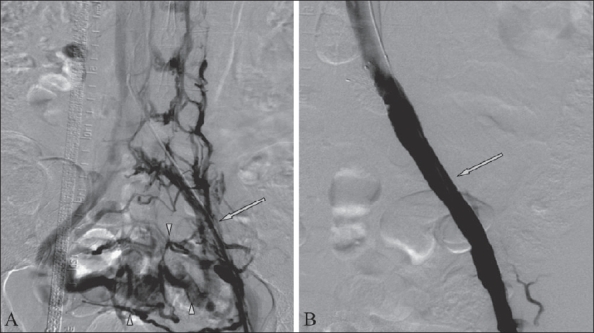
A 34-year-old patient with the May-Thurner syndrome. Venogram (A) shows occlusion (arrow) of the left EIV and CIV with a large number of collaterals (arrowheads) crossing over to the opposite side. Angioplasty was performed and three stents were deployed in the left EIV and CIV. Check venogram (B) shows good flow (arrow) in the left EIV and CIV

From 2004 to 2007, 20 patients were treated with endovascular techniques [Figure 1]. Of these, one technical failure was seen in a patient with complete occlusion of the common iliac vein, which could not be negotiated by a guide wire. Four patients with lower limb disease were treated with thromboaspiration [Figure 2]. Six patients with lower limb and two patients with upper limb disease were treated with thrombolysis. Seven patients with lower limb disease and one patient with upper limb disease were treated with angioplasty. Stents were placed in six patients with lower limb disease [Figure 3] and one patient with upper limb disease.

**Figure 4:**
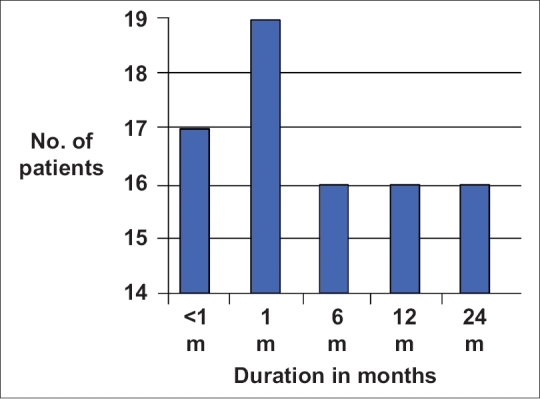
Bar diagram shows the success rate of the endovascular procedures in the management of venous disease

One-month post-procedure 17 of 19 patients remained patent, while two patients had occlusion, which was recanalized with angioplasty. At 6-month post-procedure, of the 19 patients treated, 16 remained patent, while two recanalized stents reoccluded along with the occlusion of one more stent [Figure 4].

On review at 1-year and 2-year post-therapy, of the 19 patients treated, 16 had developed no complications and showed good symptomatic improvement. The success rate in patients treated for upper limb disease was 100% and in those with lower limb disease was 81.25%.

## Discussion

Endovascular management of venous disease is a rapidly emerging alternative to conventional medical and surgical therapy for the treatment of symptomatic DVT or venous stenosis, as in the May-Thurner syndrome.

The high degree of technical success that we had, 100% in the upper limbs and 81.25% in the lower limbs, is compatible with results reported by other investigators.[9] As in our study, the usual reason for failure is the inability to catheterize a tight stenosis or chronic thrombus.

Systemic administration of IV thrombolytics is aimed at reducing the hypercoagulable component of the Virchow's triad, which in turn produces thrombolysis and thrombus removal. However, the local penetration of the thrombolytic agent into the thrombus is limited, leading to a poor success rate. Percutaneous catheter-directed thrombolysis (CDT) allows direct, local penetration of the thrombolytic agent into the thrombus and along with mechanical thrombolysis is more effective in thrombus removal and thus prevention of PE. In addition, the endovascular approach allows treatment of any associated venous stenosis by angioplasty or stent placement. Patients receiving oral anticoagulants show significant thrombolysis only in 12% of cases, while those receiving systemic IV anticoagulants have a 30% rate of thrombolysis as compared to a rate of 80% in those treated with percutaneous CDT.[12]

Patients with DVT who were earlier considered for limb amputation due to poor response to non-endovascular therapies are now seen to benefit to a great extent from percutaneous CDT. Various studies have shown a high rate of limb salvage in these patients, who present with phlegmasia cerulea dolens.[13] Surgical thrombectomy is usually the last resort in patients with a high risk of hemorrhagic complications. However, results with surgical management are not satisfactory and incomplete thrombus removal, recurrent DVT and an increased incidence of systemic complications are known.[14]

### Complications and contraindications

Candidates for thrombolysis usually have other co-morbid conditions such as hypertension, diabetes, ischemic heart disease, and bleeding disorders. The major contraindications to thrombolysis are active internal bleeding, arteriovenous malformations, recent stroke, malignancy (particularly with the possibility for brain metastases), severe uncontrolled hypertension, known bleeding diathesis, myocardial infarction due to aortic dissection and allergy to the thrombolytic agent. Recent gastro-intestinal hemorrhage, allergy to contrast material, significant liver dysfunction, renal insufficiency, acute pericarditis or subacute bacterial endocarditis, high likelihood of left heart thrombus (e.g. mitral stenosis with atrial fibrillation), diabetic retinopathy and recent arterial puncture or surgery are relative contraindications.

The various complications encountered include pulmonary thromboembolism, bleeding, septicemia, re-occlusion and malposition of stents, e.g. occlusion of the great saphenous vein while placing a stent in the superficial femoral vein. The most common complication of thrombolytic therapy is minor bleeding at the site of puncture for venous access. Pulmonary embolism is a rare complication and therefore, inferior vena cava filters are not routinely recommended. Rethrombosis that occurs in a patient in less than 30 days is generally due to poor flow and insufficient anticoagulation. This is treated with either angioplasty or stent placement.

## Conclusion

Patients presenting acutely with venous disease can be treated aggressively with catheter directed thrombolysis followed by insertion of stents. Patients presenting with more chronic symptoms can be safely and successfully treated by primary endovascular reconstruction using self-expanding stents. Catheter directed thrombolysis and venous stent placement showed comparable patency rates in our study. Venous interventions were successful in reducing limb edema and improving the ability of the patient to walk longer distances without pain.
